# Network Analysis of Acupuncture Points Used in the Treatment of Low Back Pain

**DOI:** 10.1155/2013/402180

**Published:** 2013-07-16

**Authors:** Soon-Ho Lee, Chang-Eop Kim, In-Seon Lee, Won-Mo Jung, Hong-Gee Kim, Hyunchul Jang, Sang-Jeong Kim, Hyejung Lee, Hi-Joon Park, Younbyoung Chae

**Affiliations:** ^1^Acupuncture and Meridian Science Research Center, College of Korean Medicine, Kyung Hee University, 1 Hoegi-dong, Dongdaemungu, Seoul 130-701, Republic of Korea; ^2^Biomedical Knowledge Engineering Laboratory, BK21 College of Dentistry, Seoul National University, Seoul 110-744, Republic of Korea; ^3^Department of Physiology, College of Medicine, Seoul National University, Seoul 110-744, Republic of Korea; ^4^Department of Brain Cognitive Engineering, Korea University, Seoul 136-713, Republic of Korea; ^5^Informatics Development & Management Group, Korea Institute of Oriental Medicine, Daejeon 305-390, Republic of Korea

## Abstract

*Background*. The appropriate selection of acupoints is fundamental to obtain a therapeutic effect from clinical acupuncture. *Objective*. Using a network analysis method, we investigated the acupoints that are combined to treat low back pain (LBP). *Methods*. To analyze the patterns of the combinations of acupoints, we used acupoint information from clinical trials to calculate the modified mutual information (MI) value, integrated these data, and visualized the network. *Results*. Based on the highest MI values, we found two different types of acupoint pairs used in the treatment of LBP: pairs of distant acupoints and pairs of local acupoints. Using modular analysis, we found that three acupoint modules were applied in the treatment of LBP: local acupoints, distant acupoints along the meridian, and distant acupoints based on the symptom differentiations. *Conclusion*. Using the modified MI technique, we provide a systematic framework for the acupoint combination network, and reveal how the technique of acupoint combination is used in the treatment of LBP. Application of this knowledge in acupuncture research may help clarify the mechanisms underlying acupuncture treatment at the systems level, bridging the gap between traditional medicine and modern science.

## 1. Introduction

According to traditional Asian medicine, the human body has more than 360 acupoints. Numerous combinations of these acupoints are used clinically. Ancient acupuncture practitioners discovered the therapeutic properties of acupoints and their effects not only on a particular internal organ, but also at distant parts of the body. The meridian system is a systematic order of empirical knowledge that functions as the basis of acupuncture treatment [1]. It has been reported that acupoints could exert synergistic and antagonistic actions [2, 3]. The selection of appropriate acupoint combinations is the first step in ensuring the therapeutic effect of acupuncture [4]. Practitioners select acupoints based on three basic principles: (1) local acupoints near the area where symptoms occur, (2) distant acupoints along the meridian, and (3) distant acupoints based on symptom differentiation [2].

Network science is a systems science method that is now used to construct holistic systems in diverse fields, such as the natural sciences, social sciences, and humanities [5]. Emerging trends in this new analytical science, which is based largely on graph theory, have been rapidly transferred to studies of network organization [6]. Graph theory is a natural system that can ensure the exact mathematical representation of complex networks, by representing a complex network as a graph. Network analyses use graph theory: a network is defined as a set of nodes (or vertices) and edges (or lines) between them [6, 7]. How is it possible to systematically observe the principles of acupuncture using network science? We should start by considering the similarity between the meridian system and a network. 

The meridian system is a holistic medical system that focuses on functioning at the whole-body level, and has its own theoretical framework [1, 8]. Abundant clinical data are available regarding acupuncture treatment, which is based on the holistic philosophy of traditional Asian medicine [9, 10]. The meridian system is one part of this philosophy; it consists of lines that connect surface points, which are called acupoints. Although the positions of acupoints vary according to different theories, approximately 360 acupoints are associated with 12 principal meridians. These acupoints can be considered nodes, and the meridians can be considered the edges connecting the nodes. The principles of systems biology, which is mainly associated with regulating the body's internal environment to maintain stable conditions, are closely related to those of traditional Asian medicine [11, 12]. A recent study used complex network analysis to examine the selection of acupoints for different symptoms; it matched the symptoms that every acupoint was used to treat and produced different distribution patterns [13]. Recently, Wu et al. [14] used complex network analysis to clarify the regularities and mechanisms of a reasonable combination of acupoints. Although such a bottom-up approach might seem at odds with the holistic concept of the meridian system, new research methods that incorporate the holistic features of the meridian system are urgently needed for its modernization.

In this study, we applied network analysis to determine how acupoints have been used together in combination for the treatment of low back pain (LBP). To analyze patterns in these combinations of acupoints, we calculated the modified mutual information (MI) value, integrated the data, and visualized the network based on acupoint-related information extracted from clinical trials for LBP.

## 2. Methods

### 2.1. Sources of Data about Acupoints for Low Back Pain

Previously, we analyzed the patterns of acupoints that were combined to treat LBP in 53 studies (28 Korean, 25 international; see [15]). The 33 most commonly used acupoints (frequency > 5%) were included in the present analysis of the patterns used in the acupuncture treatment for LBP.

### 2.2. Acupoint Combination Network Construction

To determine the extent of acupoint combinations used for treating LBP among each pair of the 33 acupoints, we calculated the modified MI values as described by [16], where the MI value between acupoints *x* and *y* was defined as MI(*x*, *y*) = *P*(*x*, *y*)∗ln⁡⁡(*P*(*x*, *y*)/*P*(*x*)∗*P*(*y*)), where *P*(*x*, *y*) = ∑_*i*=1_^*m*^ *I*(*x*, *y*, *i*)/*m* is the coincidence frequency of *x* and *y* and *P*(*x*) = ∑_*i*=1_^*m*^ *I*(*x*, *i*)/*m* is the frequency of acupoint *x*. This formula is the same for *P*(*y*) · *I*(*x*, *y*, *i*) is the indicator function of *x* and *y*, showing whether acupoints *x* and *y* were used together in study *i* and *m* is the total number of studies investigated. An MI matrix (33 × 33) was constructed to represent the acupoint combination network, where *a*_*ij*_ indicates the MI value between acupoints *i* and *j*.

### 2.3. Binarization of MI Matrix

To make the network tractable for analyses, the MI matrix was converted into binary adjacency matrices with only 1 or 0 as elements. While the binarizing process is commonly used for network analyses, this must be done with care to avoid misrepresenting the network by applying a specific threshold [17, 18]. We constructed adjacency matrices by applying various thresholds *R*  (0.001 ≤ *R* ≤ 0.999, in 0.001 increments). This prevented increased numbers of spurious edges and decreased the edge densities of the network, ultimately fragmenting the network into components that are inappropriate for graphtheory analyses. The maximum *R* retaining a fully connected network was 0.022. At this threshold, the edge density was 0.39. We performed all subsequent network analyses for multiple binary networks with edge densities ranging from 0.39–0.50, which retained possibly strong edges only, while the network remained fully connected. The resulting binary networks were very similar; the data are shown only for the network with an edge density of 0.40.

### 2.4. Network Analysis

Network analysis was performed using Brain Connectivity Toolbox (BCT, http://www.brain-connectivity-toolbox.net) [19]. To investigate the modular structure of the acupoint combination network, an optimization algorithm that maximizes the number of within-group edges and minimizes the number of between-group edges [20] was applied to the binarized matrix. The maximum modularity (*Q*), which quantifies the strength of the modular structure, is defined as *Q* = (1/4*l*)∑_*ij*_(*a*_*ij*_ − *k*_*i*_ *k*_*j*_/2*l*)*δ*_*ij*_, where *l* is the total number of the edges in the network; *a*_*ij*_ = 1 if node *i* and *j* are connected and 0 otherwise; *k*_*i*_ and *k*_*j*_ are the degree of each node; and *δ*_*ij*_ = 1 if *i* and *j* belong to the same module and 0 otherwise. We measured the degree and betweenness centralities of every node in each network. The degree centrality and betweenness centrality of node *i* are defined as the number of edges belonging to *i* and the fraction of shortest paths between any pair of nodes in the network passing through node *i*, respectively. The degree centrality is represented as the number of nodes to which a focal node is connected, and measures the involvement of the node in the network [21]. The betweenness centrality is a measure of the centrality of a node in a network and is equal to the number of shortest paths from all vertices to all others that pass through that node [22, 23].

### 2.5. Network Visualization

Networks were visualized using Cytoscape 2.8.3 (http://www.cytoscape.org/). We used two forms to visualize the networks: a circular layout based on modularity analysis and a force-directed layout to show the degree and betweenness centrality analysis. A circular layout places all nodes on a single circle and connects them with edges, while a force-directed layout basically uses dynamic theory [24].

## 3. Results

### 3.1. Acupoint Combination Network for Low Back Pain

This study used a modified MI technique to extract the relationships among the 33 acupoints. We examined all possible pairs and identified the most frequently used acupoint pairs. From the 13 highest MI values (>0.17), we found two different types of pair used to treat LBP (Table 1): pairs of local acupoints (BL24-BL25, BL31-BL33, BL31-BL34, BL33-BL34, BL32-GB30, BL24-BL26, BL23-BL25, BL23-BL32, and GB30-BL24) and pairs of distant acupoints (BL40-GB34, GB34-ST36, and BL40-BL60).

**Figure 1:**
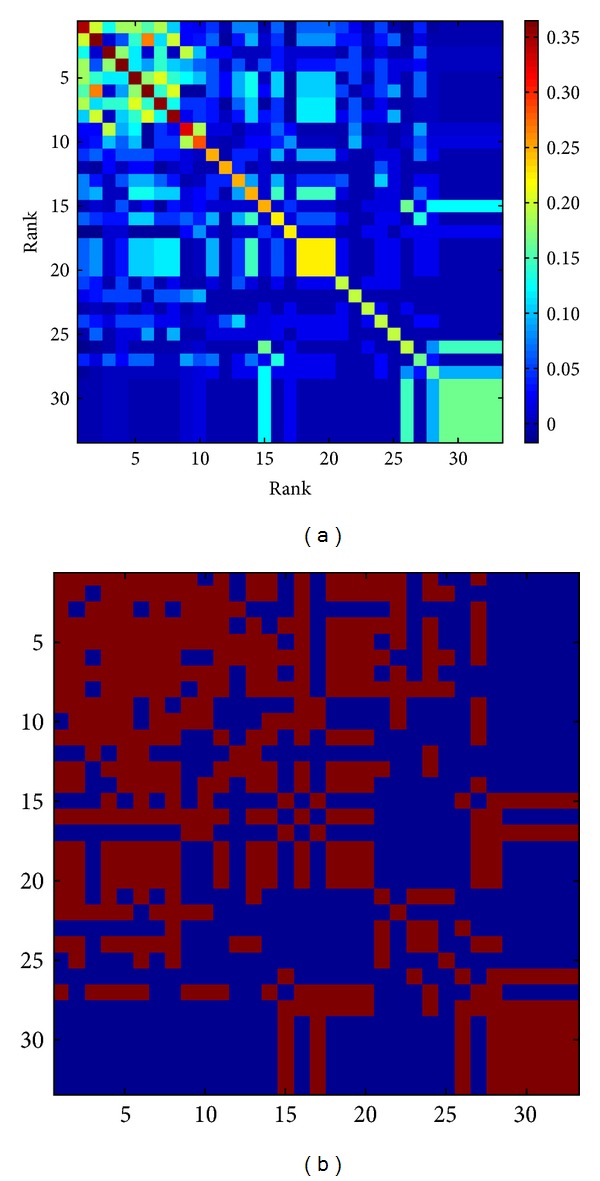
(a) The MI matrix for acupoint combinations used to treat LBP. The coordinates indicate the order of the main acupoints used for treating LBP. The distance-based mutual information model explains the relationships between all pairs of the 33 acupoints. (b) Binarized MI matrix for the acupoint pairs (threshold value = 0.031). Pairs exceeding the threshold were scored 1 and are shown in red. Pairs below the threshold were scored 0 and these pixels are in blue.

The acupoints ranked 18 to 20, that is, BL31, BL32, and BL33, had the highest MI values (Figure 1(a); yellow in the middle). The acupoints ranked 29 to 33, that is, LU8, SP3, HT8, SP2, and KI10, had relatively high MI values (Figure 1(a); light green in the right bottom). These acupoints originally ranked lower, but they were frequently used in combination, thereby increasing their MI values.

### 3.2. Modularity Analysis

**Figure 2:**
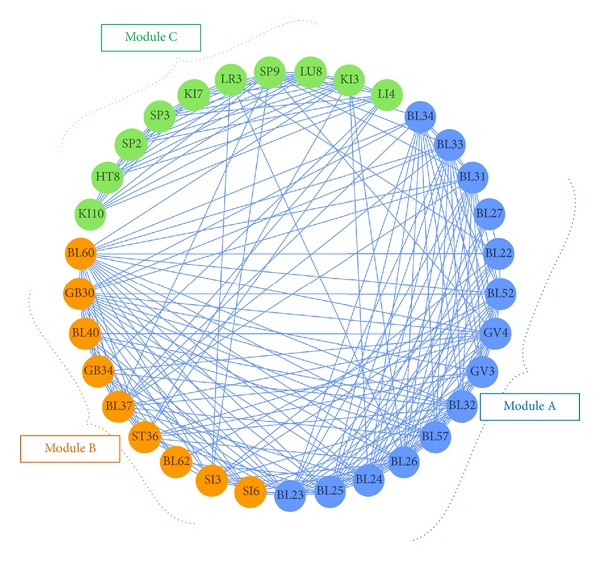
The network model based on module analysis, showing the grouped acupoints. Three modules were identified and are shown in this circular form: Module A (blue nodes), acupoints BL23, BL25, BL24, BL26, BL57, BL32, GV3, GV4, BL52, BL22, BL27, BL31, BL33, and BL34; Module B (orange nodes), acupoints BL60, GB30, BL40, GB34, BL37, ST36, BL62, SI3, and SI6; and Module C (green nodes), acupoints LI4, KI3, LU8, SP9, LR3, KI7, SP3, SP2, HT8, and KI10.

To identify patterns in the combinations of acupoints used to treat LBP, we performed a modularity analysis and illustrated the modular structure using a circular layout (Figure 2). We applied an optimization algorithm that maximizes the number of within-group edges and minimizes the number of between-group edges and thereby categorized the 33 acupoints into three acupoint modules. Module A included BL23, BL25, BL24, BL26, BL57, BL32, GV3, GV4, BL52, BL22, BL27, BL31, BL33, and BL34 (Figure 2, blue nodes). Module B included BL60, GB30, BL40, GB34, BL37, ST36, BL62, SI3, and SI6 (Figure 2, orange nodes). Module C included LI4, KI3, LU8, SP9, LR3, KI7, SP3, SP2, HT8, and KI10 (Figure 2, green nodes). Based on the characteristics of the acupoints used to treat LBP, we called Module A the Local Acupoints group, Module B the Distant Acupoints group (Type I: Distant acupoints along the meridian), and Module C the Distant Acupoints group (Type II: Distant acupoints based on symptom differentiation).

### 3.3. Network Analysis

**Figure 3:**
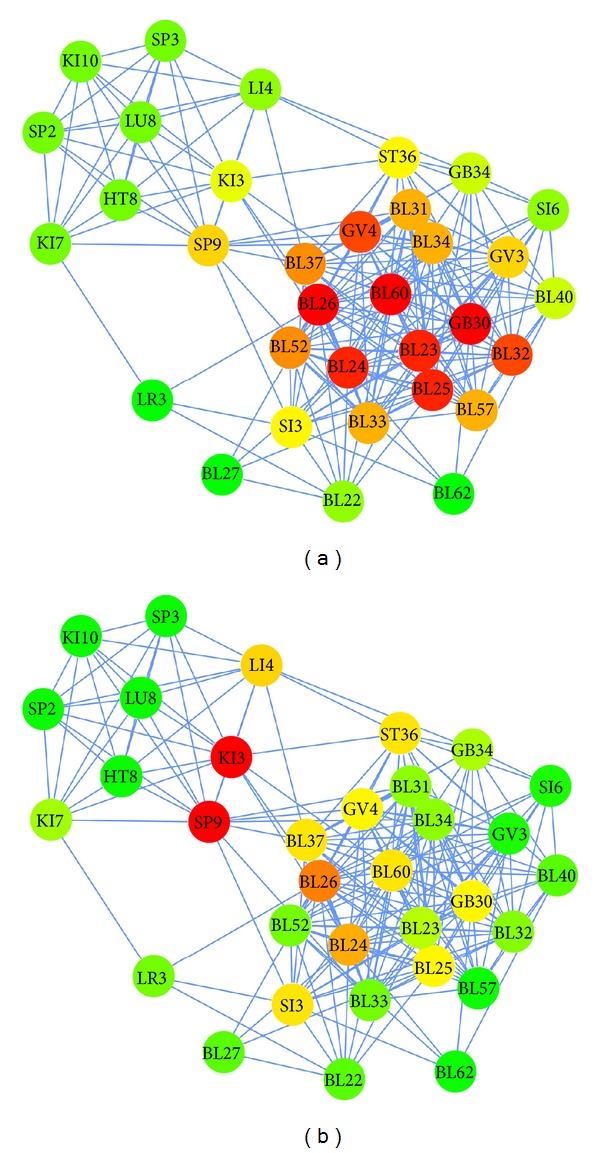
Network models showing degree centrality (a) and betweenness centrality (b). Unlike Figure 2, which groups similar acupoints as modules, this figure separates each acupoint and connects each pair with edges. In both figures, red nodes indicate a high degree of betweenness centrality, green nodes indicate a low value, and yellow nodes indicate an intermediate value.

Degree centrality analysis is useful when detecting acupoints generally used together. Nodes BL23, BL24, BL25, BL26, BL60, and GB30 had many edges, which can be explained by their frequent use in treating LBP (Figure 3(a)).  Figure 3(b), which describes the betweenness centrality of each acupoint, had a different color distribution compared to Figure 3(a). Although some acupoints have few edges, they still appear as red nodes, indicating that they have higher betweenness centrality values. Acupoints KI3 and SP9 had the highest betweenness centrality values, suggesting that these points play an important role in connecting the vertices to all others that pass through that node. We also analyzed the frequency, degree centrality, and betweenness centrality of each acupoint used in the treatment of LBP (Table 2). 

## 4. Discussion

Many patients visit traditional Asian medical clinics for acupuncture treatment. In this study, we examined what principles practitioners use to select acupoints to treat a given disease. We analyzed patterns in the combinations of acupoints used to treat LBP in clinical trials. First, we visualized the distribution of acupoint pairs based on MI values, and then we analyzed these using three different network analysis methods. These methods allowed us to identify which acupoints are frequently used together to treat LBP, and which acupoint combinations are grouped.

An MI value expresses the frequency at which a pair is used together and is defined as the intersection proportion of each proportion. Figure 1(a) is a matrix that shows MI values; the areas with high MI values are easily seen. In some ways, but not all, this result is similar to frequency: high-frequency points have large MI values. We identified which acupoints were chosen together to treat LBP, based on MI values. Ranks 18 to 20, that is, acupoints BL31, BL32, and BL33, had relatively higher MI values (Figure 1(a), yellow in the middle). These acupoints, known as the Eight Foramen Acupoints (located in the sacral foramen), are commonly used to treat LBP [25]. Additionally, acupoints LU8, SP3, HT8, SP2, and KI10 are usually used together, as shown in the MI matrix. These acupoints are the major components of the Saam Acupuncture and Eight Constitution Acupuncture regimens based on Five Phase theory and are commonly used together to treat LBP [10]. For a more elaborate explanation, we extracted the MI values listed in Table 1. The table lists the two different types of pairs of acupoints used in the treatment of LBP. One group consists of pairs between distant acupoints (e.g., BL40-GB34, GB34-ST36, and BL40-BL60), while the others are local acupoints close to the symptomatic area. The combinations involving distant acupoints might be derived from the basic principle of selecting acupoints along a meridian.

In Figure 2, the nodes are arranged around the circumference of a circle. Module A includes acupoints BL23, BL25, BL24, BL26, BL57, BL32, GV3, GV4, BL52, BL22, BL27, BL31, BL33, and BL34. All of these acupoints are on the* Bladder meridian* and the* Governing vessel*, especially at the low back position of these meridians. In traditional Asian medicine, these are considered “local acupoints,” and a local acupoint is the original and most basic choice for treatment. Module B includes acupoints BL60, GB30, BL40, GB34, BL37, ST36, BL62, SI3, and SI6. Most of these are on the* Bladder *and *Gallbladder meridians*. However, these differ from those in Module A in that the nodes are on the limbs. These are so-called “distant acupoints” and are located in positions that cause pain through the same meridian on the opposite side of the body or the adjacent meridian. These points function as a “remote control panel” of the meridian Qi. Finally, Module C includes acupoints LI4, KI3, LU8, SP9, LR3, KI7, SP3, SP2, HT8, and KI10. These acupoints are generally positioned on the limbs but not on the* Bladder meridian*. They are used to treat LBP based on symptom differentiation. These findings about the three acupoint modules confirmed that the three basic principles of acupuncture regimens (local acupoints, distant acupoints along the meridian, and distant acupoints based on symptom differentiation) are also applied in the treatment of LBP [2].

In the network analysis, we analyzed the degree and betweenness centrality. The results are shown in Figure 3. In Figure 3(a), nodes with a high degree value are colored red, while ones with low values are green. Acupoints BL23, BL25, BL60, GB30, and BL26 appear to be widely used to treat LBP. While the degree refers to the strength of one node in connections, betweenness centrality implies the rate of connectivity between a node and other indirect nodes. As shown in Figure 3(b), acupoints KI3 and SP9 are nodes that connect Module C (distant acupoints based on symptom differentiation) to Module A (local acupoints) or Module B (distant acupoints along the meridian). These points function as “hubs” that connect to different types of node. Therefore, we postulate that acupoints KI3 and SP9 play a crucial role in the interconnection between the local and distant acupoints modules.

In summary, this study clarified which acupoints are frequently used together to treat LBP. The modified MI technique provides a systematic framework for the acupoint combination network and helps reveal how the technique of acupoint combination is used in the treatment of LBP. This study is the first to apply network science to explore unique theories related to the meridian system, and the findings provide new insights into acupoint selection based on biomedical information. Combining network science with clinical data about acupuncture treatment will help facilitate the evaluation of the therapeutic effects of acupuncture, and its possible mechanisms at the system level, bridging the gap between traditional medicine and modern science.

## Figures and Tables

**Table 1:** Acupoint pairs with high mutual information values.

Acupoint pairs	MI value
(L) BL24-BL25	0.2678
(L) BL31–BL33	0.2227
(L) BL31–BL34	0.2227
(L) BL33-BL34	0.2227
(L) BL32–GB30	0.2069
(L) BL24–BL26	0.2069
(L) BL25-BL26	0.2023
(L) BL23–BL25	0.2018
(D) BL40–GB34	0.1966
(L) BL23–BL32	0.1909
(D) GB34–ST36	0.1900
(D) BL40–BL60	0.1786
(L) GB30–BL24	0.1786

Acupoint pairs with mutual information values > 0.17 are shown. (L) indicates local acupoint pairs and (D) indicates distant acupoint pairs.

**Table 2:** Frequency, degree centrality, and betweenness centrality of acupoints.

Rank	Acupoint	Frequency (%)	Degree	Betweenness centrality
1	BL23	50.9	19	0.01747
2	BL25	43.4	19	0.02772
3	BL40	32.1	11	0.00809
4	BL60	32.1	20	0.04194
5	GB30	32.1	20	0.02363
6	BL24	32.1	19	0.05881
7	BL32	28.3	18	0.01249
8	BL26	28.3	20	0.07760
9	GB34	20.8	11	0.01639
10	ST36	15.1	13	0.03434
11	BL57	11.3	15	0.00338
12	BL62	11.3	5	0.00108
13	BL52	11.3	16	0.01067
14	GV3	11.3	14	0.00245
15	KI3	11.3	12	0.11167
16	GV4	9.4	18	0.02451
17	LI4	9.4	10	0.04310
18	BL31	9.4	15	0.01335
19	BL33	9.4	15	0.01335
20	BL34	9.4	15	0.01335
21	BL22	7.5	9	0.00827
22	SI6	7.5	9	0.00131
23	LR3	7.5	4	0.01094
24	SI3	7.5	13	0.04064
25	BL27	7.5	4	0.00000
26	KI7	7.5	8	0.01540
27	BL37	5.7	16	0.03787
28	SP9	5.7	14	0.13137
29	LU8	5.7	8	0.00065
30	SP3	5.7	8	0.00065
31	HT8	5.7	8	0.00065
32	SP2	5.7	8	0.00065
33	KI10	5.7	8	0.00065

## References

[1] Yin C-S, Koh H-G (2005). What’s the original concept of meridian and acupuncture point in oriental medicine?—a perspective of medical history. *Uisahak*.

[2] Chen Y-R, Zhu J, Song J-S, She Y-F (2012). Discussion on point selection and compatibility of acupuncture formula. *Zhongguo Zhen Jiu*.

[3] Zhong F, Zeng F, Zheng H, Ren C, Jin R-J, Li Y (2011). Present study on antagonistic effect of acupoints compatibility. *Zhongguo Zhen Jiu*.

[4] Wang H, Liang FX (2012). Thoughts and prospects of research on acupoints compatibility. *Zhongguo Zhen Jiu*.

[5] Börner K, Sanyal S, Vespignani A (2007). Network science. *Annual Review of Information Science and Technology*.

[6] Bullmore E, Sporns O (2009). Complex brain networks: graph theoretical analysis of structural and functional systems. *Nature Reviews Neuroscience*.

[7] Boccaletti S, Latora V, Moreno Y, Chavez M, Hwang D-U (2006). Complex networks: structure and dynamics. *Physics Reports*.

[8] Jung W (2010). The origin of Jingmai: through the investigation into some important hypotheses. *Korean Journal of Medical History*.

[9] Vickers AJ, Cronin AM, Maschino AC (2012). Acupuncture for chronic pain: individual patient data meta-analysis. *Archives of Internal Medicine*.

[10] Yin C, Park H-J, Chae Y (2007). Korean acupuncture: the individualized and practical acupuncture. *Neurological Research*.

[11] Van Der Greef J (2011). Perspective: all systems go. *Nature*.

[12] Kitano H (2002). Systems biology: a brief overview. *Science*.

[13] Wang Y-Y, Lin F, Jiang Z-L (2011). Pattern of acupoint selection based on complex network analysis technique. *Zhongguo Zhen Jiu*.

[14] Wu QF, Zhang CS, Chen Q, Yu SG (2012). On feasibility of researching acupoint combination by using complex network analysis techniques. *Zhen Ci Yan Jiu*.

[15] Lee I, Jo H, Lee S (2012). Systematic review of selection of acupuncture points for lower back pain. *Korean Journal of Acupuncture*.

[16] Li S, Zhang B, Jiang D, Wei Y, Zhang N (2010). Herb network construction and co-module analysis for uncovering the combination rule of traditional Chinese herbal formulae. *BMC Bioinformatics*.

[17] Wig GS, Schlaggar BL, Petersen SE (2011). Concepts and principles in the analysis of brain networks. *Annals of the New York Academy of Sciences*.

[18] Power JD, Fair DA, Schlaggar BL, Petersen SE (2010). The development of human functional brain networks. *Neuron*.

[19] Rubinov M, Sporns O (2010). Complex network measures of brain connectivity: uses and interpretations. *NeuroImage*.

[20] Newman ME (2006). Finding community structure in networks using the eigenvectors of matrices. *Physical Review E*.

[21] Opsahl T, Agneessens F, Skvoretz J (2010). Node centrality in weighted networks: generalizing degree and shortest paths. *Social Networks*.

[22] Barthélemy M (2004). Betweenness centrality in large complex networks. *European Physical Journal B*.

[23] Goh K-I, Oh E, Kahng B, Kim D (2003). Betweenness centrality correlation in social networks. *Physical Review E*.

[24] Han K, Ju B-H (2003). A fast layout algorithm for protein interaction networks. *Bioinformatics*.

[25] Lund I, Lundeberg T, Lönnberg L, Svensson E (2006). Decrease of pregnant women’s pelvic pain after acupuncture: a randomized controlled single-blind study. *Acta Obstetricia et Gynecologica Scandinavica*.

